# The Effect of D-Tagatose on Fructose Absorption in a Rat Model

**DOI:** 10.4172/2329-6631.1000111

**Published:** 2013-09-10

**Authors:** Jarrod Williams, Michael Spitnale, Robert Lodder

**Affiliations:** Department of Pharmaceutical Sciences, College of Pharmacy, University of Kentucky, USA

**Keywords:** D-tagatose, Diabetic medication, Oral absorption, Fructose, Oral gavage

## Abstract

D-tagatose is in development as a medication for the treatment of type 2 diabetes. The effect of oral D-tagatose on the absorption of D-fructose was assessed when co-administered in this study. In the pilot study, male Sprague-Dawley rats were fed C14 labeled fructose and glucose concomitantly to establish dose levels for the treatment group of rats fed C14 labeled fructose together with D-tagatose. Rats were administered 0, 600, 2000, 6000, or 12000 mg/kg of D-tagatose along with 2000 mg/kg of fructose. Blood samples were taken over 60 minutes and were assessed using scintillation counting. 600, 2000, and 6000 mg/kg of D-tagatose decreased fructose absorption by 1%, 26%, and 30% respectively (12000 mg/kg group was stopped short of completion due to intolerance) as measured by AUC of scintillation counts. The 600 and 2000 mg/kg of D-tagatose groups showed no difference in plasma glucose concentrations compared to placebo while a rise in glucose was seen in the 6000 mg/kg of D-tagatose groups. The results indicate that D-tagatose may be useful in reducing fructose absorption, which could lead to a beneficial outcome.

## Introduction

A naturally occurring epimer of fructose, D-tagatose is present in small amounts in many dairy products, such as milk, numerous cheeses, and certain kinds of yogurts [1,2]. Interest in D-tagatose as a sweetening alternative to conventional sugar manifested from the finding that it provided no available energy when fed to rats [3], yet it maintains a sweetness equivalent to 92% that of sucrose [4]. D-tagatose was subsequently examined and granted US patent approval in 1988, as a low calorie full bulk sweetening agent [5].

The majority of research in the following years revolved around elucidating the toxicity profile of the substance, with the aim of pursuing FDA approval as a food additive. Multiple *in vitro* and *in vivo* studies were completed with the results establishing that D-tagatose was not genotoxic [6], no evidence of treatment-related effects were seen at low doses, and at high doses, the transient toxicity of soft stools consistent with osmotic diarrhea (from incomplete absorption of the carbohydrate) occurred [7,8]. Human studies then commenced with results closely mimicking that of the animal models [9,10]. A decade of human, animal and other toxicity and safety data led to D-tagatose gaining “Generally Recognized As Safe” (GRAS) approval by the FDA [11], allowing its use in food and beverages in limited amounts. No incidence of toxicity has been reported to date from usage in food and beverages. Following this, the European Union (EU) allowed D-tagatose as a ‘novel food ingredient’, with no restrictions on the amount in which it can be used [12].

Interest in the molecule as a pharmaceutical agent began as a result of discovering it to have antidiabetic properties in animal models [13]. Previous GRAS approval allowed prompt entry of D-tagatose into human studies as an antihyperglycemic agent. Early results indicated that there was no change in glucose or insulin levels, following oral administration of D-tagatose in the fasted state [14]. Even more interestingly, D-tagatose showed the ability to blunt the rise in blood glucose, when given prior to oral glucose intake [15]. These studies conducted at the University Of Maryland School Of Medicine and subsequently confirmed at the Research Department of Nutrition, Copenhagen [16] led to the consensus that D-tagatose held the potential of becoming an adjunct therapy for patients with Type II *Diabetes mellitus*.

In 2010, a phase II dose ranging study over 6 months and a phase III efficacy study over 12 months were completed using D-tagatose to reduce HbA1c in 161 and 494 patients, respectively, in the US and in India (clinicaltrials.gov identifier NCT00961662 and NCT00955747). The results showed statistically significant reductions in HbA1c compared to placebo, with the only adverse effects being of the GI variety, consistent with prior studies. The exciting results of these studies offered promise for D-tagatose as a diabetic medication and warranted further examination into how D-tagatose achieves its effects, and how it interacts with other components of the human diet, especially other carbohydrates [17].

Previous work in multiple labs has explored the variable absorption of different carbohydrates when administered alone, or in conjunction with other carbohydrates. Truswell et al. [18] determined that fructose exhibits a saturable effect upon oral absorption. When given 50 g orally of pure fructose, 58 of 100 subjects were classified as poor absorbers, while only 19 of these previous poor absorbers were unable to properly absorb 25 g of pure fructose. Interestingly, when glucose was given with 50 g of fructose, the number of poor absorbers of fructose was cut in half. This study was confirmed years later in a rat model, in which researchers at the University of California claimed the mechanism of the effect is due to the disaccharidase-related transport system [19]. Prieto et al. [20] then demonstrated that the alterations in absorption between glucose alone, fructose alone, and the combination of glucose and fructose, in fact manifested as changes in glycemia and insulin responses.

In light of these findings and the increasing prevalence of fructose in modern foods, it became important to distinguish the consequences of D-tagatose on fructose absorption, if it were to be given therapeutically as an antihyperglycemic medication. The present study aimed to provide new information D-tagatose consumption with fructose through administration of ^14^C labeled fructose and D-tagatose concomitantly, and comparing the radioactivity present in plasma samples collected at various time points to that of fructose monotherapy in a rat model. We first completed a pilot study using [^14^C]-fructose with glucose, to establish the viability of our procedure to measure changes in carbohydrate absorption, and subsequent plasma levels, and to derive the amount of radioactivity that would be needed in the fructose batch to obtain interpretable scintillation count data. From the results of this initial study, we aimed to establish the effect that a graduated change in concentration of D-tagatose would have on absorption of 2 g/kg of fructose, with a secondary aim of determining how the resulting absorption would manifest in terms of blood glucose.

## Materials and Methods

### Animals

Male Sprague-Dawley FVC (FVC=femoral venous catheter) rats from Harlan weighing 275 to 300 g were used for these studies. The Sprague-Dawley FVC rats had femoral vein cannulas (implanted by the animal vendor) tunneled under the skin that exit superior to the scapula. Catheters were checked for patency and maintained with heparin+glycerol (1000 IU/ml). Patency checks were conducted on the day of receipt, and at regular intervals until use. Locking solutions were composed of an equal mixture of USP grade glycerol and heparin (1000 IU/ml). Rats were housed in solid bottom cages with bedding in ventilated stainless-steel racks, and were individually caged to prevent cage mates from gnawing on cannulae. The rats were placed in a normal light cycle, 12 hours light and 12 hours dark, and were housed for 3 to 7 days in a vivarium prior to testing. Animals were conditioned to hooded restrainers for approximately 15 min on days (−5 to −3) and (−3 to −1), prior to the start of the study.

All animal care and procedures of the study were performed in compliance with the U.S. Department of Agriculture’s (USDA) Animal Welfare Act (9 CFR Parts 1, 2, and 3); the Guide for the Care and Use of Laboratory Animals, Institute of Laboratory Animal Resources, National Academy Press, Washington, D.C., 1996; and the National Institutes of Health, Office of Laboratory Animal Welfare.

### Plasma [^14^C]-fructose measurement following oral administration of a bolus [^14^C]-fructose/glucose preparation

Two fructose/glucose dosing solutions containing either 250 uCi/g or 83.5 uCi/g [^14^C]-fructose were prepared to deliver 2 g/kg of each sugar via oral gavages. Radiolabeled fructose was purchased from American Radiolabeled Chemicals, St. Louis, MO. Two additional dosing solutions containing either 250 uCi/g or 83.5 uCi/g [^14^C]-fructose were prepared to deliver 2 g/kg fructose alone *via* oral gavages. Four groups of rats (8 rats per group) were dosed as follows: Group 1–2000 mg/kg [^14^C]-fructose (250 uCi/g); Group 2–2000 mg/kg [^14^C]-fructose (250 uCi/g)+2000 mg/kg glucose; Group 3–2000 mg/kg [^14^C]-fructose (83.5 uCi/g); Group 4–2000 mg/kg [^14^C]-fructose (83.5 uCi/g)+2000 mg/kg glucose. At 0, 1, 3, 5, 10, 15, 30, 45, and 60 minutes time points, whole blood was obtained from the venous catheter, measured for total glucose, centrifuged, and plasma retained for scintillation counting of ^14^C.

### Effect of tagatose on plasma [^14^C]-fructose absorption following oral administration of a bolus [^14^C]-fructose/tagatose preparation

A fructose dosing solution containing 250 uCi/g [^14^C]-fructose was prepared to deliver 2 g/kg of fructose alone or with either vehicle, 0.6 g/kg, 2 g/kg, 6 g/kg, or 15 g/kg D-tagatose *via* oral gavages. Five groups of rats (8 rats per group) were dosed as follows: Group 1–2000 mg/kg [^14^C]-fructose (250 uCi/g); Group 2–2000 mg/kg [^14^C]-fructose (250 uCi/g)+tagatose 600 mg/kg; Group 3–2000 mg/kg [^14^C]-fructose (250 uCi/g)+tagatose 2000 mg/kg; Group 4–2000 mg/kg [^14^C]-fructose (250 uCi/g)+tagatose 6000 mg/kg; Group 5–2000 mg/kg [^14^C]-fructose (250 uCi/g)+tagatose 12000 mg/kg. At 0, 1, 3, 5, 10, 15, 30, 45, 60 minute time points whole blood was obtained from the venous catheter, measured for total glucose, centrifuged, and plasma retained for scintillation counting of ^14^C.

### Sample analysis

Blood samples were analyzed for total glucose using a MediSense™ Precision PCx^®^ Glucometer (Model M6012-0014) with MediSense™ Precision PCx^®^ Glucose Test Strips (Model 99565-01). Scintillation counting for ^14^C was conducted on 10–30 uL of plasma in 10 mL of Fisher ScintiSafe Gel (SX24-5) scintillation fluid, using a Beckman scintillation counter (Model LS6500).

## Results

**Figure 1:**
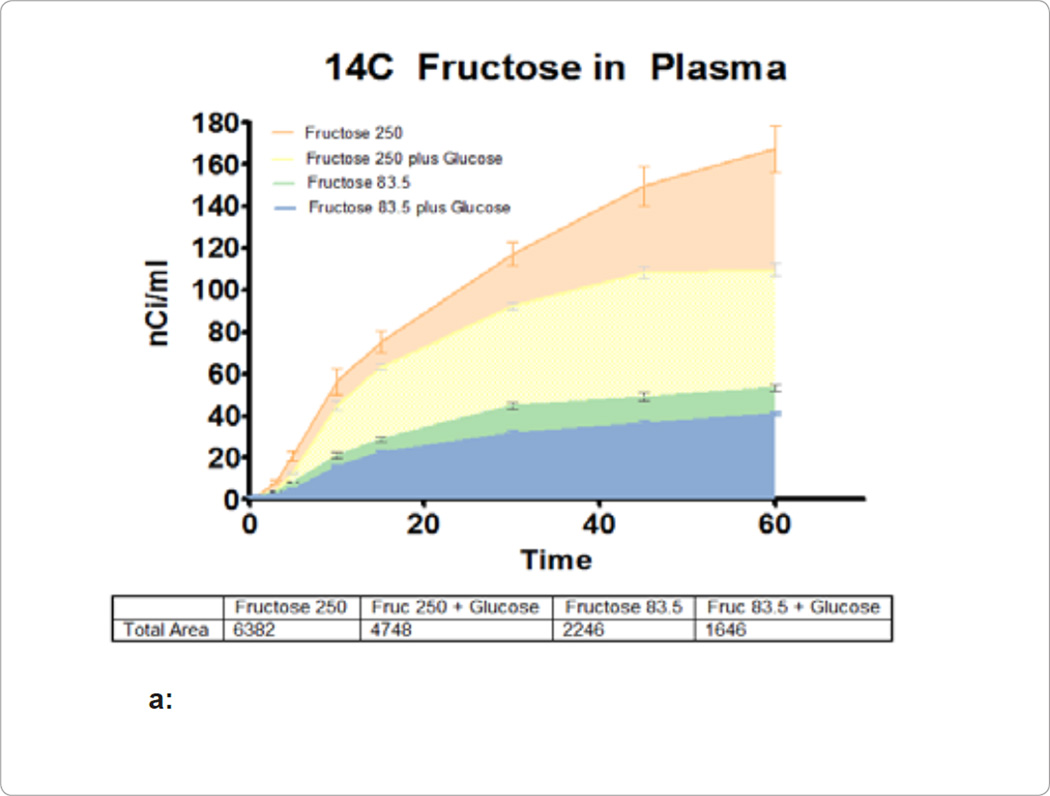

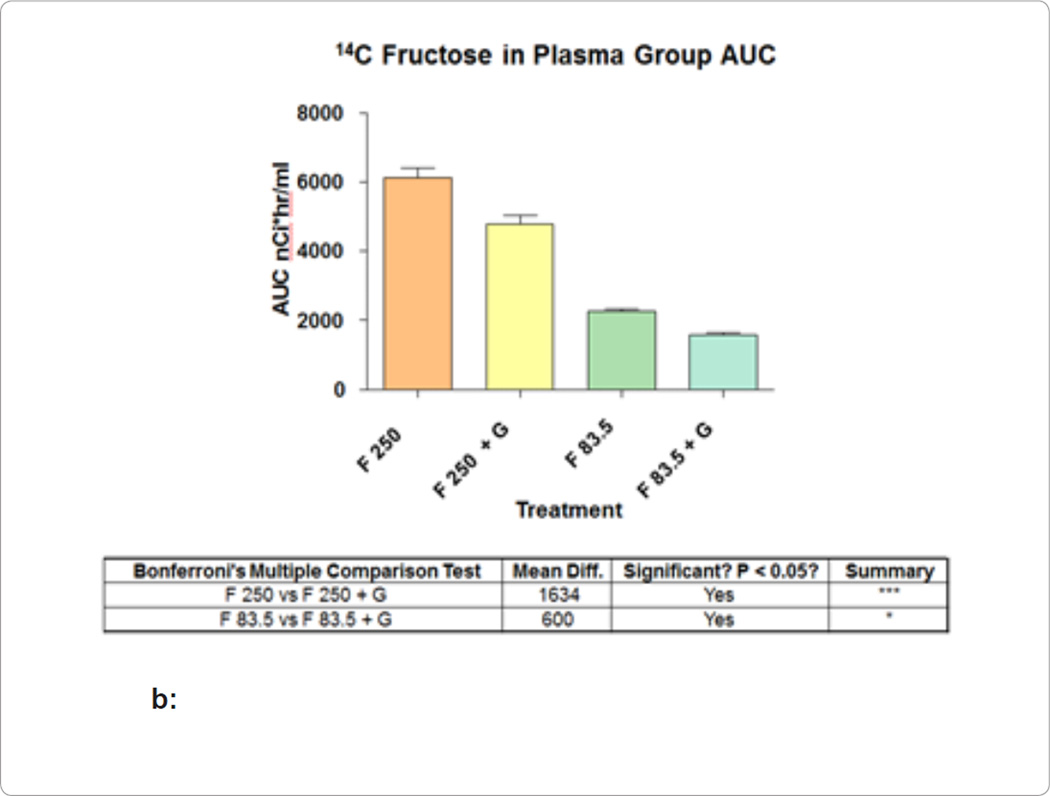
**a:** Radioactivity measured in nCi/mL is plotted over the 1 hour study for each of the 4 arms. The AUC for each group is also included in the table below the figure. **b:** Display of the relative differences between AUC of the different treatment arm measured in nCi*hr/ml. The table below the figure gives the numerical values for these differences.

The pilot study aimed to set the level of radioactivity to be used later in the fructose/tagatose portion by use of a well known pairing (glucose: fructose), and to verify that changes in systemic carbohydrate levels could be measured accurately using this procedure. Figure 1A show the accumulation of radioactivity in the plasma at various time points. The AUC for each group is superimposed over each other, and the amount of activity in nCi/ml over the 60 minute study is displayed. Addition of glucose to fructose in equal amounts (2 mg/kg weight of rat) resulted in a decrease in plasma fructose of 26% in the 250 uCi/g group and 27% in the 83.5 uCi/g group. Figure 1B compares the effect glucose had on fructose absorption. Administration of glucose with fructose resulted in a mean difference of 1634 and 600 nCi·hr/ml in the 250 and 83.5 uCi/g of fructose groups, respectively, and reached significance as calculated by the Bonferroni’s Multiple Comparison Test.

**Figure 2:**
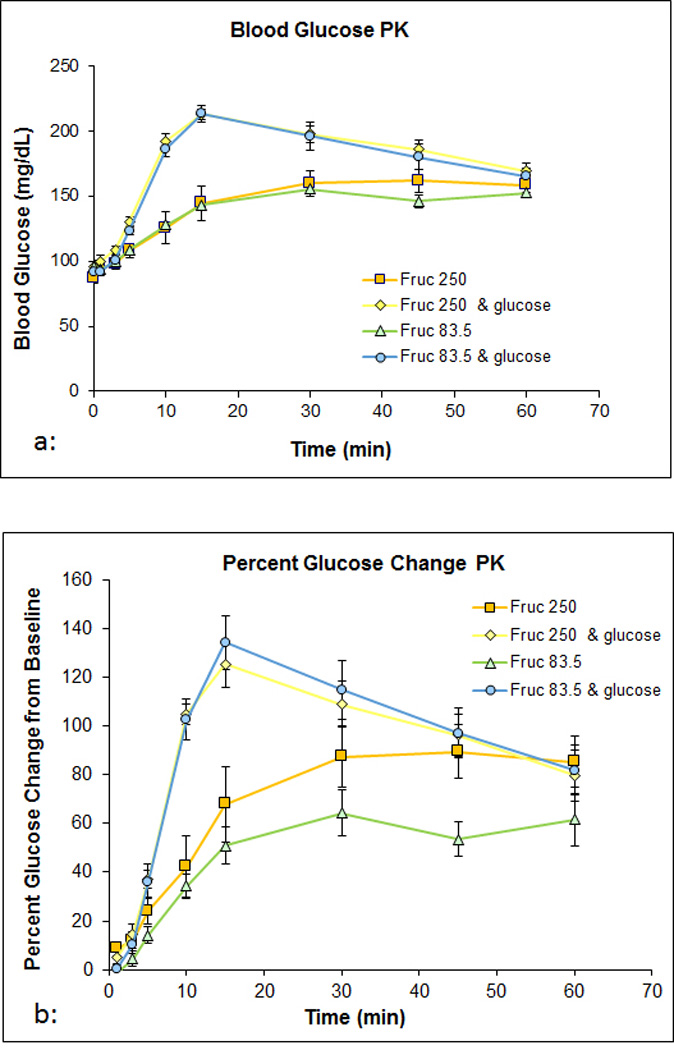
**a:** Displays the blood glucose concentrations given in mg/dl at each time point during treatment. **b:** Displays the blood glucose readings as a percentage change from baseline.

Figure 2A compares the different treatment arms with respect to blood glucose throughout the study. Administration of fructose *via* oral gavages resulted in an increase in glucose levels in all arms of the study. Figure 2B shows the change in glucose concentration from baseline (taken at time 0 before administration of treatment). Oral fructose resulted in a change in blood glucose that peaked at a concentration of 80 mg/dl in the fructose alone groups. When glucose was given with fructose, the amount of glucose in the blood peaked near 140 mg/dl. Additionally, the time to peak glucose concentration occurred at the 30 or 45 minute time point in the fructose alone arms, and at the 15 minute time point in the fructose/glucose co-administration groups.

**Figure 3:**
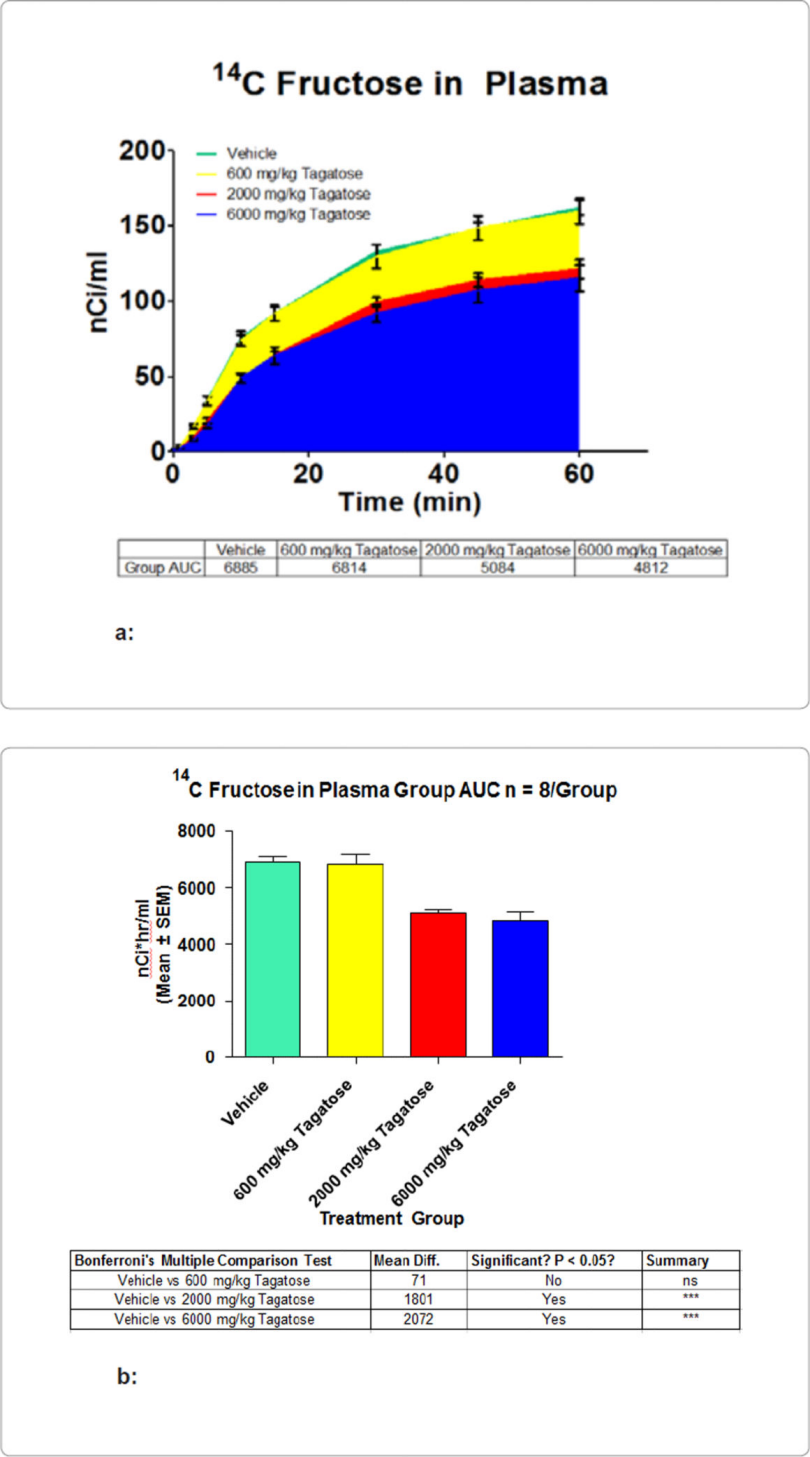
**a:** Radioactivity measured in nCi/mL is plotted over the 1 hour study for each of the 4 arms. The AUC for each group is also included in the table below the figure. **b:** Display of the relative differences between AUC of the different treatment arm measured in nCi*hr/ml. The table below the figure gives the numerical values for these differences.

The results of the pilot study established that 250 uCi of radioactivity per gram of fructose would provide adequate scintillation counts to measure a change in fructose absorbance with co-administration of another carbohydrate. The following figures document the simultaneous intake of [^14^C]-fructose with 250 uCi/g of radioactivity administered at 2 g/kg of body weight with vehicle, 0.6 g/kg, 2 g/kg, and 6 g/kg of D-tagatose *via* oral gavages. Figure 3A shows the effect that D-tagatose had on the AUC of radioactivity from [^14^C]-fructose in the plasma over the 60 minutes of the study. Figure 3A represents this by overlapping the graphs of AUC from the different treatment arms. Figure 3B shows that 600, 2000, and 6000 mg of D-tagatose per kg of body weight decreased the AUC of fructose absorption by 71 (1% decrease), 1801 (26% decrease), and 2072 (30% decrease) nCi·hr/ml, respectively. The 2000 and 6000 mg/kg doses reached significance, whereas the 600 mg/kg group did not (the 12000 mg/kg group was stopped early in the study due to intolerance and 1 fatality).

**Figure 4:**
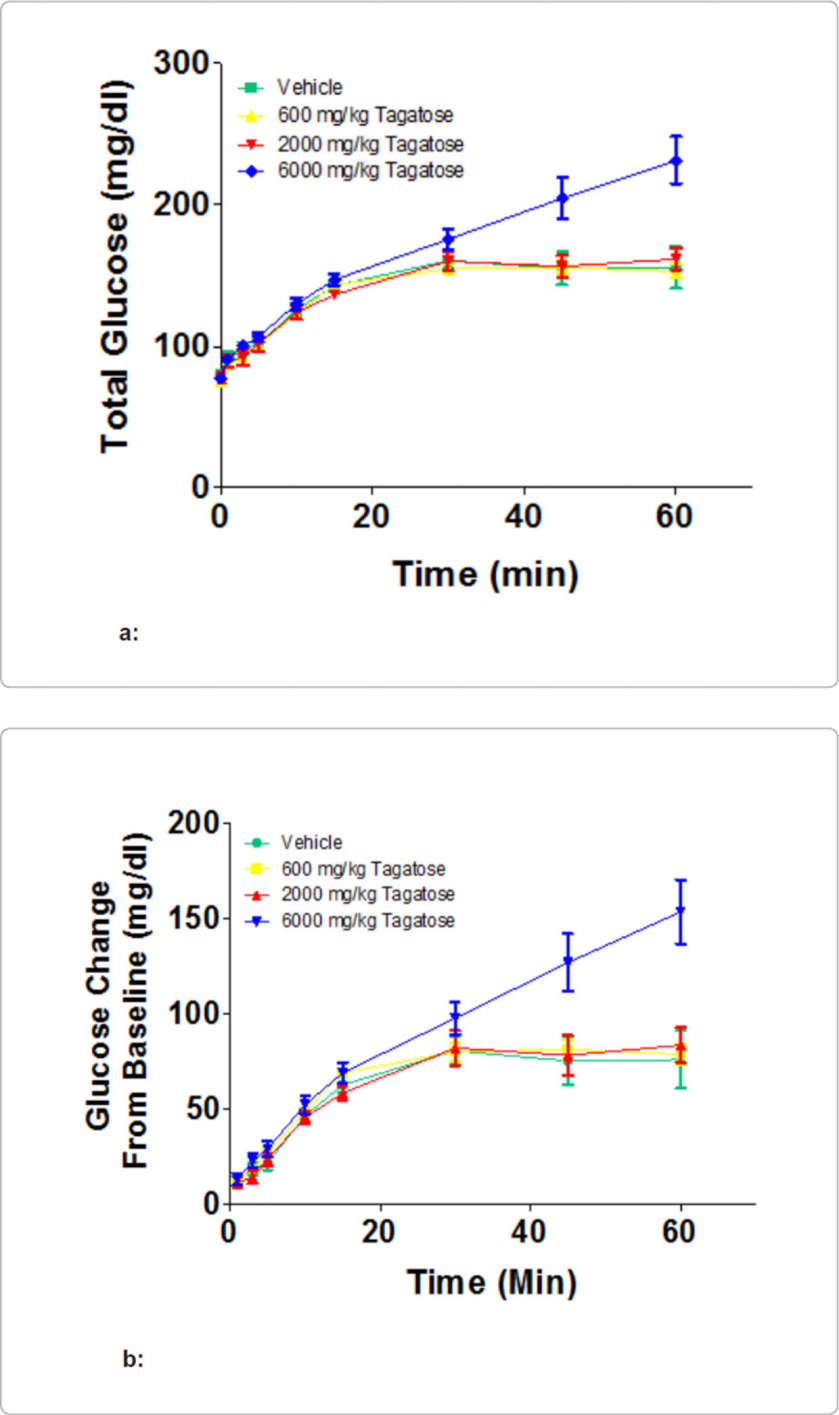
**a:** Displays the blood glucose concentrations given in mg/dl at each time point during treatment. **b:** Displays the blood glucose readings as a percentage change from baseline.

Figures 4A and 4B illustrates the effect the effect of the administration of 2 mg of fructose per kg of body weight along with either vehicle, 600, 2000, or 6000 mg/kg of D-tagatose on blood glucose. The 600, and 2000 mg/kg of D-tagatose groups produced blood glucose levels that were no different than with administration of fructose alone (vehicle). At 6000 mg/kg, an elevation in blood glucose was seen at the 30 minute time point that continued to rise, reaching 232 mg/dl at the last sampling (minute 60). This represented nearly a 75 mg/dl difference from the vehicle, 600 and 2000 mg/kg groups.

## Discussion

The study at hand consisted of two phases. The aim of the pilot portion was twofold: (1) to determine the level of radioactivity in fructose that needed to be present in order to produce readily measurable scintillation counts in plasma samples, and (2) to validate that the procedure we were using could determine changes in the systemic levels of carbohydrates with statistical significance. The second portion of the protocol held the answers to the questions that motivated us to pursue the study. Here, we used the exercise validated in the pilot to elucidate the effect that D-tagatose would have on absorption of fructose co-administered with it in an *in vivo* rat model. We also established the effect that the absorbed fructose had on glycemia, or more importantly, the ability of D-tagatose to blunt the glycemic effect of fructose.

The pilot study determined that a radioactivity level of 250 uCi/g of fructose was sufficient to accurately measure the amount of fructose in the plasma after oral administration *via* oral gavages. The amount of fructose present in plasma when administered with equal amounts of glucose was reduced compared to fructose monotherapy. Additionally, fructose administration resulted in hyperglycemia in all arms of the study. Both these findings may appear to be controversial in light of other literature [18–20], yet there are several reasons this conclusion might be unwarranted. First, the pilot study was not designed for external validity with previous studies, only as a pilot study to lay the groundwork for the actual second part of the fructose/tagatose study to follow. For instance, the pilot study lacked a sham group (receiving the catheter but no active substance). Because of this, it is impossible to determine the effect that the procedure itself had on absorption. Boudry et al. [21] established in a study conducted in 2007, that stress induced to rats can cause an upregulation of GLUT2 at the apical membrane of the brush border of the intestines, and cause a shift from absorption of glucose through SGLT1 to absorption through GLUT2. GLUT2 is a known transporter of glucose, fructose, and galactose in high amounts (low selectivity, high capacity) [22]. Even though the animals were preconditioned to the procedure, it is possible that the stress of the procedure itself, or the placement of the catheter, caused an upregulation in GLUT2 at the luminal membrane, which has been documented to occur in the span of minutes [23]. Stress could cause increased absorption of fructose during monotherapy and a reduction in fructose absorption in the presence of glucose, as it competes for transit *via* the same GLUT2 transporter that is facilitating a major portion of the fructose absorption. Additionally, the lack of a glucose-alone treatment group inhibits us from comparing the ability that fructose itself had to decrease glycemia, as shown in some of the studies mentioned previously. Again, the pilot study was not designed to be compared to these studies, simply to provide a reference for the second study of the effect that D-tagatose would have on glycemia, when administered with fructose (compared to fructose alone).

In the second portion of the study, we showed that D-tagatose was able to decrease the amount of fructose absorbed when co-administered *via* oral gavage. This reduction was most prevalent at the 2000 mg/kg dose. This reduction did not seem to be strongly dose dependent. The 6000 mg/kg dose of D-tagatose was only slightly better at inhibiting fructose absorption. We believe this is simply a situation of saturation of carbohydrates. There was no run-in period to acclimate the rats to D-tagatose or fructose. In fact, the 12000 mg/kg dosing group had to be stopped due to fatality. At an acute dose of 6000 mg/kg group and up, and even with 2 g/kg of fructose, the rats seem to be in a state of malabsorption, characterized by osmotic diarrhea and symptoms of overt stress. D-tagatose is an epimer of fructose, and the rats responded to the protocol as if they had been given a single dose of fructose that grossly exceeded their ability to absorb it. Previous studies have shown fructose malabsorption to be around 2 g/kg, so with the addition of D-tagatose, all of our arms were above this range. We also suspect that the stress of malabsorption and correlating symptoms explains the rise in blood glucose in the 6000 mg/kg dosing group.

The mechanism by which D-tagatose is able to reduce HbA1c in diabetic patients is not fully established. The phase 2 and 3 clinical trials showed that with a 2-week run-in period malabsorption could be controlled even with high doses of D-tagatose three times daily, while maintaining HbA1c reduction. In the 1-year phase 3 trial, HbA1c continued to drop month by month throughout the trial with no signs of tachyphylaxis. The present study provides another small piece of the puzzle, and a future study will determine the ultimate fate of the fructose.

When AUC is used to measure exposure of a drug, integration of peak area is usually performed until drug is completely eliminated from the body. As we were measuring a radioisotope of carbon, it was impossible to do this in our study, because we could not establish when the carbon would have been metabolized from fructose into another molecule. Therefore, the absorption of fructose may have simply been delayed and would have shown up, had we performed a different study over a longer period. A full metabolic study of the fructose in a given dose in the presence of D-tagatose will answer many such questions. Elucidation of the mechanisms of action of D-tagatose may help to optimize an interesting candidate for anti-diabetic therapy, or isolate a population of diabetics in which this drug could be maximally effective.
